# S13-3 Translating visionary ideas into real solutions across VARCITIES' Pilot Cities

**DOI:** 10.1093/eurpub/ckad133.065

**Published:** 2023-09-11

**Authors:** Sara Van Rompaey

**Affiliations:** E2ARC

## Abstract

VARCITIES has identified seven European municipalities as the Pilot Cities of the project. Together we will implement integrated and sustainable initiatives that increase the health and well-being of citizens, supporting both municipal actions and local SMEs in meeting credible opportunities to grow and generate revenues.

Each pilot city has recognized a large-scale site for co-designing and implementing local actions and has adopted an innovative cross-sectoral approach by combining urban digital transformation with nature-based actions. This innovative solution's implementation is consistent with the increase in citizens' awareness, respect for public spaces, and the integration of green spaces into everyday life. Developing a healthy green mindset for citizens and improving economic opportunities through green-digital strategies, drives the implementation of additional actions towards becoming a healthier, more sustainable and equitable city. The development process of these visionary ideas into feasible actions is approached by following a bottom-up planning and co-design participatory process involving local stakeholders, assuming a “multiple benefits” perspective, and also addressing social issues and cultural diversion. Over 25 visionary solutions were implemented across the pilot sites focusing on various target groups from the children, senior citizens, patients of hospitals, people with physical or mental disabilities, to all citizens of the cities.

The visionary solutions vary from interconnection among sport, recreational and therapeutic facilities, to analysis and monitoring of psychological and physiological well-being for elderly people and people affected by Alzheimer, or educating and engaging citizens to level-up their awareness of climate change and the importance of biodiversity. They are focused on enhancing the overall effectiveness of green spaces on the quality of life, and physical and mental health of citizens as well as helping to educate them on those topics. The visionary solutions can be duplicated and implemented by other cities in order to promote continuous improvement of health and living quality globally.

